# Tuning Natural Killer Cell Anti-multiple Myeloma Reactivity by Targeting Inhibitory Signaling *via* KIR and NKG2A

**DOI:** 10.3389/fimmu.2018.02848

**Published:** 2018-12-04

**Authors:** Niken M. Mahaweni, Femke A. I. Ehlers, Gerard M. J. Bos, Lotte Wieten

**Affiliations:** ^1^Division of Hematology, Department of Internal Medicine, Maastricht University Medical Center+, Maastricht, Netherlands; ^2^GROW School for Oncology and Developmental Biology, Maastricht University, Maastricht, Netherlands; ^3^Tissue Typing Laboratory, Department of Transplantation Immunology, Maastricht University Medical Center+, Maastricht, Netherlands

**Keywords:** NK cell, NKG2A, KIR, HLA class I, HLA-E, multiple myeloma, immunotherapy

## Abstract

Natural killer (NK) cells are attractive candidates for allogeneic cell-based immunotherapy due to their potent antitumor effector function and good safety profile. NK cells express killer immunoglobulin-like receptors (KIRs) and the NKG2A receptor important for NK cells education as well as providing inhibitory signals upon encountering HLA-expressing target cells. Multiple myeloma (MM) is an example of a tumor expressing relatively high levels of HLA molecules. In this review, we discuss the functional relevance of inhibitory KIRs and NKG2A for NK cells anti-MM response and strategies to lower these inhibitory signaling to enhance clinical efficacy of allogeneic NK cells in MM.

## Introduction

Over the past years, NK cells became popular candidates for immunotherapy against cancer due to their unique combination of a potent anti-tumor effector function and a very good safety profile (1). The capacity of NK cells to discriminate healthy- from diseased cells creates the opportunity to safely use NK cells in the allogeneic setting and to maximally benefit from their anti-tumor potential while not risking development of graft vs. host pathology. In the allogeneic setting, this latter feature is a great benefit over strategies using conventional T cells. Although both T cells and NK cells exploit major histocompatibility (MHC) molecules for immune surveillance, they do it in an intrinsically different manner. T cell activation occurs upon interaction between the T-cell receptor (TCR) and a foreign MHC-peptide complex which, in an allogeneic MHC mismatched setting, easily results in graft vs. host disease (GVHD). NK cells, on the other hand, rather sense the absence of MHC molecules, a phenomenon called “missing-self recognition” described first in the 1990's by Ljunggren and Karre (2). Even in the absence of MHC molecules, NK cells do not attack healthy cells because for activation a sufficient level of activating signals, provided by viral- or stress proteins, is required and these signals are usually not sufficiently present on healthy cells (3).

NK cells can sense self vs. missing-self *via* receptors belonging to the killer immunoglobulin-like receptor (KIR) family and NKG2A. In this review, we will provide an overview of the functional relevance of KIR and NKG2A for the anti-tumor response of NK cells in an allogeneic setting. We will specifically address the role of allogeneic NK cells in multiple myeloma (MM), a hematological malignancy characterized by the expansion of malignant plasma cells in the bone marrow. To date, MM remains incurable despite the greatly improved clinical perspective due to novel immunomodulatory agents like lenalidomide and pomalidomide and highly promising antibodies like daratumumab (anti-CD38) and elotuzumab (anti-CS-1/SLAMF7). Given their excellent safety and feasibility profiles, NK cells are interesting candidates to combine with these novel agents to enhance clinical efficacy and to ultimate achieve curative treatment for MM patients.

## Killer Immunoglobulin-Like Receptors (KIRs) Biology

The KIR family consists of activating- and inhibitory receptors. Activating family members are characterized by a short cytoplasmic ITAM activating signaling domain and are called KIRxDS. Inhibitory family members have a long and inhibitory ITIM domain and are named KIRxDL. Both the activating and the inhibitory KIRs have two (KIR2DSx or KIR2DLx) or three (KIR3DSx or KIR3DLx) extracellular immunoglobulin-like domains for ligand interaction. Classical human leukocyte antigen (HLA) class I molecules are the most important ligands for both the activating- and the inhibitory KIRs. The best characterized inhibitory KIRs are: KIR2DL1, binding to HLA-C group 2 (C2) alleles having a lysine at position 80; KIR2DL2/3, interacting with HLA-C group 1 (C1) alleles having an asparagine at position 80 (4–6). KIR3DL1, binding HLA-B alleles bearing a Bw4 motif as well as HLA-A^*^23/^*^24/^*^32 (7, 8). KIR3DL2 has been shown to interact with HLA-A^*^3/^*^11 (9) and HLA-F (10). The activating KIR2DS1 and KIR2DS2 have been shown to bind with C2 and C1 alleles, respectively, and KIR2DS4 interacts with subsets of HLA-C alleles and with HLA-A^*^11 (11, 12). The ligands for the other KIRs remain elusive so far.

The genes encoding the KIRs are located in the KIR gene cluster in the leukocyte receptor region on chromosome 19, and so far, 27 different KIR haplotypes have been described (http://www.imgt.org/). KIR2DL4, KIR3DL2, KIR3DL3, and KIR3DP1 are so called framework genes and are present in all the haplotypes. Based on the additional presence/absence of the other KIRs, the haplotypes can be further grouped into haplotype-A and –B. While A haplotypes express only KIR2DS4 as activating KIR and eight other KIRs (KIR2DL1, KIR2DL3, KIR2DL4, KIR3DL1, KIR3DL2, KIR3DL3, KIR2DP1, and KIR3DP1), the B haplotypes express multiple activating receptors in combination with various other *KIR* genes (13). In the population, the A to B haplotype ratio is on average 1.8:1 (14) and in most populations B/x haplotypes are more common than A/A. A study comparing KIR haplotype A and B frequencies in MM demonstrated that there was no difference in distribution between MM patients and healthy individuals (14). Moreover, analysis of KIR repertoires of 182 MM patients revealed that the genotypic presence of KIR3DS1, most pronounced in Bw4 missing patients, was associated with reduced progression free survival after autologous SCT (15). Nonetheless, further extensive studies on the influence of the KIR genetic repertoire on development and progression of MM are missing.

Further variation in KIR repertoires between individuals results from the relatively polymorphic nature of the *KIR* genes and expression differences can occur due to null/low/high expression allele variants and copy number variation (16). Furthermore, KIRs are acquired in a stochastic manner leading to intra-individual diversity in KIR receptor expression between NK cells (17). Within the A haplotype four inhibitory KIRs, namely KIR2DL1, KIR2DL3, KIR3DL1, KIR3DL2 can be expressed. A combination of cell surface expression of all four inhibitory KIRs is rarely found within one healthy individual (< 5%). Co-expression of three inhibitory KIRs occurs also in rather few NK cells (about 10%), while co-expression of 2 KIRs and expression of a single KIR occurs more frequently (30% and 35%, respectively). Functionally immature NK cells, lacking all KIRs, represent about 20% (18).

## NKG2A Receptor Biology

NK cells of healthy individuals frequently express NKG2A (20–80%) (19, 20). NKG2A expression occurs more frequently on KIR-negative NK cells and decreases as NK cells acquire KIRs (18). NKG2A is an inhibitory member of the C-type lectin-like NKG2 receptor family that also comprises the inhibitory NKG2B and the activating NKG2C/E/H receptors (21). NKG2A engages HLA-E, a non-classical HLA class I molecule constitutively expressed at low levels on the cell surface of virtually every cell. In contrast to the classical HLA class I molecules, HLA-E displays only very limited polymorphism and only two common protein variants are known (HLA-E^*^01:01 and HLA-E^*^01:03) (21). These two HLA-E allelic variants differ in one amino acid at position 107 on the alpha2 domain of the HLA-E heavy chain, and HLA-E^*^01:01 has an Arginine at position 107 while HLA-E^*^01:03 has a Glycine (22). This amino acid difference results in a higher peptide binding affinity and consequently a higher surface expression for HLA-E^*^01:03. NKG2A binds to both HLA allotypes and so far, no obvious functional differences between the two HLA-E alleles have been described (23, 24). While the KIRs are highly polymorphic, NKG2A is well conserved with only a few known polymorphism (13, 25, 26).

## NK Cell Education and Recognition of Missing Self

Inhibitory receptors for HLA play a pivotal role in the shaping of a functional NK cell repertoire. NK cells develop from the bone marrow and acquire inhibitory receptors in a stochastic manner (27). Mature NK cells can express no-, one- or a combination of inhibitory receptors. As the KIR and HLA genes are located on different chromosomes (KIR on chromosome 19 and HLA on chromosome 6) they can be inherited independently. Consequently, individuals can express KIRs for which the corresponding HLA ligand is missing. For example, an individual can express KIR3DL1 without being Bw4 positive. To warrant self-tolerance, even in the absence of a ligand, NK cells are continuously educated by their HLA environment in a process called “licensing” or “arming.” Although the mechanistic basis of this process is not fully understood, it is known that NK cell subsets expressing no inhibitory receptor or a receptor for which the HLA ligand is not endogenously expressed are hyporesponsive (28). On the other hand, NK cells expressing inhibitory receptors that can engage HLA become more responsive (29). Those so-called educated NK cells have been shown to hold higher density granules (30). Moreover, they are more potent cytokine producers and killers than non-educated NK cells (31). From previous studies it known that the more inhibitory receptors an NK cell expresses, the more potent its effector function (32, 33).

## HLA Class I and HLA-E Expression in Multiple Myeloma

Many viruses and tumors have evolved strategies to reduce HLA expression presumably to escape from CD8 T cell immunity, and educated NK cells are excellent in targeting these cells. However, while loss of expression of classical HLA class I is frequently seen in many types of cancer it is becoming more and more clear that numerous of these tumors remain positive for HLA-E (34). By doing so, these tumor cells can evade from CD8 T cells and, as the majority of the NK cells expresses NKG2A, the tumor also remains relatively protected against NK cells. Under physiological conditions, HLA-E expression is tightly linked to HLA class I expression. The reason is that HLA-E presents the leader peptides that are removed from HLA class I molecules before leaving the endoplasmic reticulum to travel to the cell surface (35). Consequently, a reduced expression of HLA class I can be expected to result in lower levels of HLA-E on the cell surface. But, apparently this is not necessarily the case, and tumors, as well as several viruses, have developed ways to maintain HLA-E expression even in the absence of HLA class I leader peptides. Why this is possible is not completely known. One option could be that HLA-E presents a TAP- and HLA class I independent peptide repertoire, as seen in TAP deficient LCL 721.221 cells (36). Alternatively, peptides from stress proteins like Hsp60 have been shown stabilize HLA-E on the cell membrane (37).

In MM, we observed that MM cell lines frequently express only low levels of HLA-E *in vitro* while HLA-E expression was much higher upon *in vivo* growth of the cells in the bone marrow of immunodeficient mice (38). Moreover, primary MM cells obtained from patients expressed relatively high levels of HLA class I as well as HLA-E (38). Furthermore, HLA expression has been shown to be related to disease status in MM, since MM cells isolated from late-stage pleural effusions expressed higher levels of HLA class I and reduced levels of activating NKG2D ligands as compared to earlier stage MM cells (39). A comparable observation was made by comparing MGUS vs. MM samples, showing higher levels of HLA class I and reduced levels of MICA on the MM samples (40). Given the presence of both HLA class I and HLA-E on MM, interfering with inhibitory signaling to lower the NK cell activation threshold, so basically creating missing-self, for NK cells in MM seems a good strategy. This could be perceived either by KIR-ligand mismatching based on genotypes in an allogeneic transplantation setting, the use of clinically available monoclonal antibodies to block KIR (e.g., lirilumumab) or NKG2A (e.g., monalizumab) (41, 42), or by agents such as the proteasome inhibitors lactacystin, bortezomib and carfilzomib that have been shown to reduce HLA class I expression in MM (43–45).

## Creating Missing-Self for Multiple Myeloma by KIR-Ligand Mismatching in the Allo-SCT Setting

**Figure 1:**
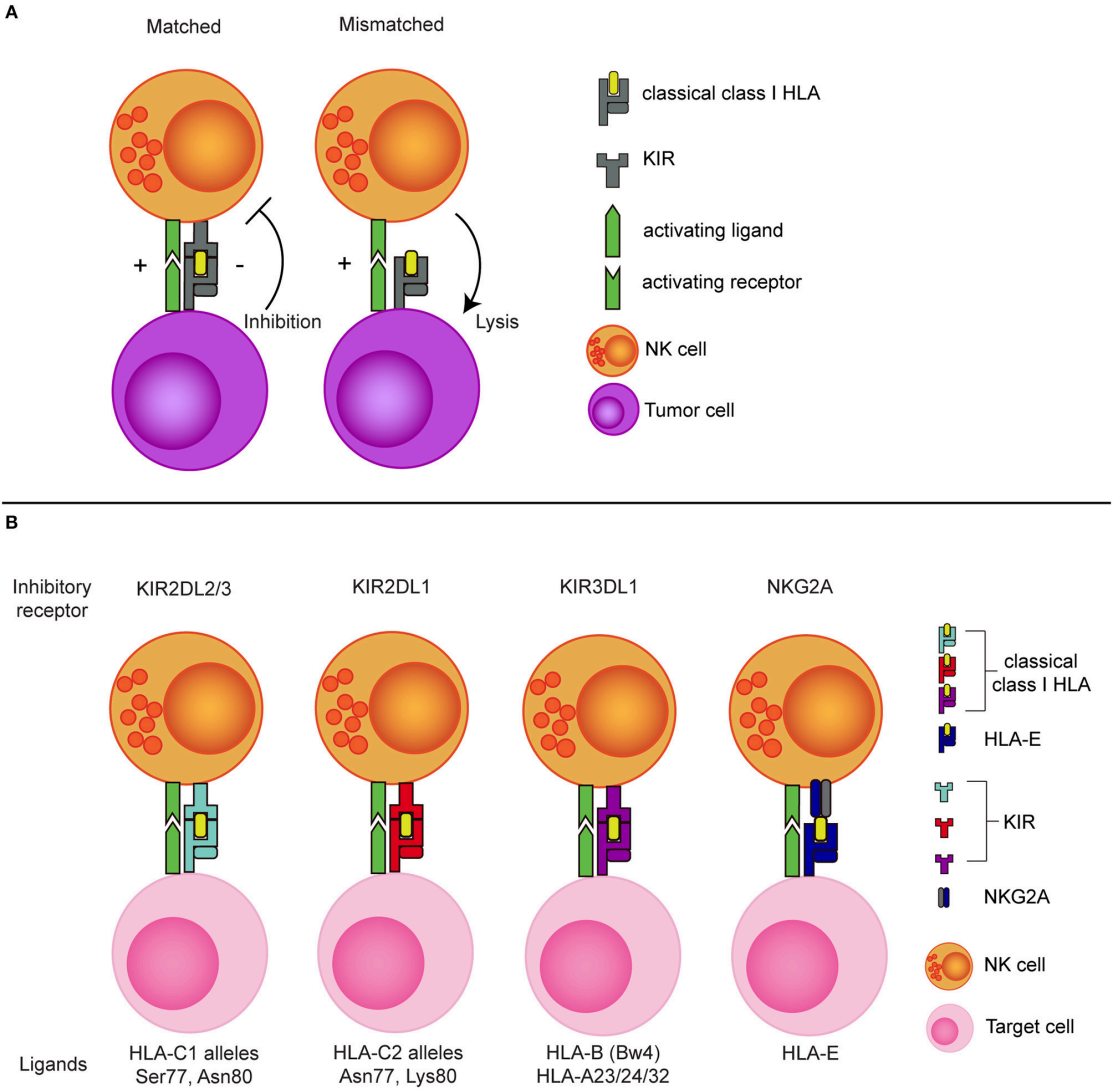
The concept of NK cell alloreactivity concept based on interaction with HLA class I. **(A)** When an inhibitory KIR binds to a “matched” classical class I HLA molecule, an NK cell receives inhibitory signal from this interaction. In the absence of the corresponding class I HLA molecule (mismatched situation), the inhibitory signal is absent, resulting in a reduced NK cell activation threshold. **(B)** Inhibitory KIRs and NKG2A and their corresponding class I HLA molecules. KIR, Killer immunoglobulin-like receptor; HLA, Human leukocyte antigen; Ser, Serine; Asn, Aspargine; Lys, Lysine.

The potential of exploiting missing-self recognition to enhance the antitumor potential of NK cells in the allogeneic setting became most evident from the ground breaking work of Ruggeri et al. showing that a so called KIR-ligand mismatch improved clinical outcome after haploidentical stem cell transplantation (haplo-SCT) in patients with acute myeloid leukemia (AML) (46, 47). In the haplo-SCT setting, patient and donor are matched based on one of the HLA haplotypes meaning that half of the HLA genes is mismatched between patient and donor. This enables incompatibility between inhibitory KIRs, expressed on the donor NK cells, and their HLA ligands on patient tumor cells. As the donor KIR-ligand mismatched NK cells in this setting will remain educated by the donor HLA background (48, 49), they can efficiently detect missing-self and mediate more potent responses against the tumor cells than the non-mismatched NK cells that receive inhibitory signals *via* HLA (Figure 1).

Only very limited data on the potential benefit of KIR-ligand mismatching in allo-SCT in MM is available. Nevertheless, Kröger et al. showed that in HLA-C mismatched unrelated donor allo-SCT, patients receiving a KIR-ligand mismatched graft had longer progression free survival than patients receiving a matched graft (50). In general, there is still no real consensus on whether or not a KIR-ligand mismatch has a clinical benefit and presumably this is highly dependent on the exact conditioning- and transplantation protocol. In contrast to KIR-HLA class I, mismatching for HLA-E and NKG2A is not an option due to the limited polymorphism of HLA-E. However, early upon reconstitution, the relatively immature NK cells express NKG2A but not KIRs and it can take up to 3 months till a fully mature KIR repertoire is present (51). As NKG2A could inhibit the anti-MM response of these reconstituting NK cells (38), it may be an interesting option to interfere with HLA-E NKG2A interaction using a monoclonal antibody like monalizumab in the context of allo-SCT.

For a long time, feasibility of haplo-SCT was limited by the high occurrence of post-transplant complications such as GVHD and infections. However, due to the recent successes of improved T cell depletion methods (e.g., by αβ-depletion) or by post-transplant administration of cyclophosphamide, haplo-SCT became a feasible approach with a good safety profile and the major advantage that a large number of donors is usually available within the family (52). To be eligible for KIR-ligand mismatched transplantation, patients should genotypically lack expression of at least one of the inhibitory KIR ligands, meaning that they should miss either HLA-C1, -C2, or –Bw4 or a combination thereof. Nonetheless, ~1/3 of the population express all three ligands (53). This was also observed in a large clinical study on leukemia and myelodysplastic syndrome (54), where around 34–36 % of the patients expressed all three ligands and the rest of the patients (64–66 %) lacked at least one ligand. In addition, to the absence of C1, C2, or Bw4 in the patient, the selected donor should express the HLA ligand that is missing in the patient to make sure the NK cells will be educated to sense the missing ligand and the corresponding KIR should be present on the cell surface. Especially for KIR3DL1 this is important to confirm, preferably by flow cytometry, as null alleles frequently occur (55).

## Creating Missing-Self for NK Cell Adoptive Transfer in Multiple Myeloma

Various research groups have shown that NK cells played a major role in the elimination of tumor cells in the allogenic setting. Nonetheless, previous studies have also shown that NK-cell numbers and effector to target ratios' are important for tumor cell clearance (56, 57). To enable infusion of high numbers of NK cells, large scale *ex vivo* epansion of NK cells for adoptive NK cell-based therapy is currently heavily investigated (58, 59).To date, several groups have infused NK cells into MM patients as a form of adoptive immunotherapy. Szmania et al. infused up to 1 × 10^8^ (per kilogram) *ex vivo* expanded NK cells derived from MM patients or haploidentical family donors into 8 high-risk relapsed MM patients (60). These NK cell infusions were well tolerated and a significant *in vivo* expansion of the NK cells was observed in two subjects. Although in five patients the NK cell infusion did not affect the disease progression, in one patient it resulted in a partial response and in another patient in a delayed disease progression. In another stud*y*, umbilical cord blood-derived NK cells were infused into 12 high-risk relapsed MM patients (61). In this study, four different doses of NK cells were administered each to three patients; 5 × 10^6^, 1 × 10^7^, 5 × 10^7^, and 1 × 10^8^ demonstrating a good safety profile a partial response in 10 patients as their best response. In the 21st month of follow up, four patients progressed or relapsed.

Given the relatively high expression of HLA-class I on the MM cell surface (38, 39, 62), selection of KIR-ligand mismatched NK cell donors could be an approach to enhance clinical anti-MM responses of infused NK cells. As the potential benefit of a KIR-ligand mismatch is not very well established in MM, we recently addressed this question in a series of *in vitro* studies in which we were especially interested in the functional relevance of KIR-ligand mismatching for highly activated NK cells. The reason for this was that most current insight in the role of KIR and NKG2A comes from studies using unactivated NK cells or from NK cells that are reconstituting after allo-SCT. The situation might be very different for the highly activated NK cells that are typically used for adoptive NK cell therapy as their activation threshold could be changed by the activation. Our studies revealed that for unactivated NK cells as well as for highly activated (1,000 U/mL IL-2) NK cells, KIR-ligand mismatched NK cells were better effector cells than KIR-ligand matched NK cells against various MM cell lines (38). This was also the case in the presence of immunosuppressive factors, like hypoxia, PGE2 and lactate, that are frequently found in tumor microenvironment (63). Even when we further potentiated the NK-cell anti-MM response *via* antibody-dependent cell-mediated cytotoxicity (ADCC), by combining NK-cells and daratumumab (anti-CD38), KIR ligand mismatched NK cells degranulated more robustly than their matched counterparts (63). Although the difference between the matched and mismatched subsets was not very large, one can anticipate that in an immunosuppressive tumor microenvironment, where the NK cell receives and integrates a multitude of inhibitory signals, reduction of any extra inhibitory signals by KIR-ligand mismatching could help to potentiate the NK cell response against HLA class I competent MM cells. As many of the currently used *ex vivo* expanded clinical NK cell products harbor very high percentages of NKG2A+ NK cells, we also evaluated the relevance of NKG2A interaction for the anti-MM response of highly activated NK cells. This showed that, at least *in vitro* and on *ex vivo* primary MM cells, the level of HLA-E on the MM cells was not sufficient to trigger potent inhibitory signaling *via* CD94-NKG2A (64). Enhanced levels of HLA-E on the MM cells, by using an HLA-E stabilizing peptide, did, however, result in inhibition *via* NKG2A, and illustrated that the expression level of HLA-E influenced the inhibitory potential of NKG2A (64). Together these data emphasize the complexity of the NK cell antitumor response. Furthermore, they suggest that creating missing self by KIR-ligand mismatching for highly activated NK cells could help to potentiate clinical efficiency while creating missing-self based on interfering with NKG2A may only be beneficial for tumors expressing high levels of HLA-E.

## Creating Missing-Self With Monoclonal Anti-KIR or Anti-NKG2A Antibodies

The use of currently clinically available antibodies that block KIR or NKG2A is an alternative interesting option to create missing-self in MM. Blocking antibodies would especially be helpful for the 30% of donors expressing all three KIR ligands. It may also be applied under conditions where NKG2A is mediating strong inhibitory effects (e.g., for tumors with very high levels of HLA-E or for unactivated NK cells).

Blocking KIR-ligand interaction using an anti-HLA antibody showed enhanced killing of primary MM by haploidentical KIR-ligand mismatched NK cells in an *in vitro* autologous transplantation setting (65). In other *in vitro* studies, the addition of IPH2101, a clinical anti-KIR antibody, increased NK cell cytotoxicity against HLA-C positive acute myeloid leukemia and lymphoma cells (66, 67). In spite of the *in vitro* successes, the clinical efficacy of the antibody still needs to be further elucidated. In a phase I clinical study in patients with relapsed/refractory MM, the IPH2101 antibody has been shown to be safe and well tolerated, however, it did not result in clear clinical responses despite an observed improvement in *in vitro* cytotoxicity against a MM cell line (41). A phase II trial with IPH2101 in patients with smoldering MM, was prematurely terminated due to lack of therapeutic benefit (68). To unravel the unexpected lack of benefit, a follow up study was performed which showed that infusion of IPH2101 had led to both reduced KIR2D surface expression on NK cells and reduced NK function (69). KIR2D removal of anti-KIR treated NK cells was mediated by trogocytosis, a mechanism by which monocytes remove antibody-bound molecules from the cell surface. These studies suggest that blocking KIR by anti-KIR antibodies could result in uneducated, hyporesponsive NK cells and subsequently in limited effects of the antibody *in vivo*. Also they illustrate that, despite its *in vitro* potential, a better understanding of how to use the blocking antibody *in vivo* is essential.

One way to improve clinical responses of IPH2101 may be by combinational therapies with drugs providing strong activating signals to the NK cells. In a phase I clinical trial with relapsed/refractory MM patients, the combination of the anti-KIR antibody with lenalidomide, an immunomodulatory agent, augmented NK cell function and resulted in objective responses (70). Moreover, combination of IPH2101 and the ADCC-triggering antibody daratumumab could also enhance NK cell cytotoxicity against MM cell lines and against primary myeloma cells *in vitro* while IPH2101 alone did not induce a significant antitumor effect in this setting (71).

Blocking NKG2A is another option to reduce inhibitory NK cell signaling aiming to improve the effector function of either endogenous NK cells or of donor NK cell in allo-SCT or adoptive transfer settings. In a preclinical mouse study, infusion of NKG2A+ NK cells mediated anti-leukemia effects when NK cells were pre-treated with an anti-NKG2A antibody and rescued the mice from developing leukemia (42). In another *in vitro* preclinical study, blocking NKG2A with the anti-NKG2A antibody monalizumab could restore the cytotoxic potential of NK cells derived from patients with chronic lymphocytic leukemia (72). However, thus far completed clinical trials testing safety and efficacy of monalizumab in MM patients are not available.

## Future Perspective

To further enhance the NK-cell antitumor response, novel combination strategies are currently being explored and also there it may be relevant to evaluate the additive value of interfering with inhibitory signaling *via* HLA. Examples of such strategies are combinations with antibodies (monoclonal, bi- or tri-specific) targeting tumor-associated or –specific antigens to trigger ADCC. Also, genetic modification of NK cells during e*x vivo* NK cell expansion is frequently explored as a novel way to improve NK function for instance by creating NK cells expressing chimeric-antigen receptors (CAR) to potently trigger NK cell activation. Another interesting option is the combination of haplo-SCT and infusion of a high number of highly activated NK cells from the same donor. This combination would bypass the drawbacks of slow reconstitution of mature NK cells (up to 2–6 months) in haplo SCT (73, 74, 75) and lack of persistence for *ex vivo* expanded NK cells as they are short lived and not clonally expand upon activation (76). The combination setting would have the best of both worlds. First, the adoptively transferred NK cells could be manipulated during *ex vivo* expansion and they can mediate their potent antitumor effects in the first lymphopenic period after haplo-SCT while they simultaneously contribute to protection from viral infections. Second, the NK cells that reconstitute from the donor stem cells will provide persistence of donor NK cells. Third, the process of donor selection for both procedures needs to only be done once. The use of haplo-SCT in Europe is increasing since 2005 (77). Our group recently finished a phase I study performing haplo-SCT in MM, now continued as a phase II study (NL49476.000.14), which can serve as platform for haplo-SCT and NK-infusion combination therapy in MM. At MD Anderson such a combination study has already been in patients with AML showing feasibility and better disease free survival and less infections (78).

Studies with sufficient power to demonstrate a potential clinical relevance of creating missing-self are currently lacking in MM. Moreover, since the benefit of interfering with inhibitory HLA-induced signaling seems to depend on the exact transplantation protocol, the activation status of the NK cell, and on the input via other activating- or inhibitory receptors, it will be important to test the clinical relevance for NK cells receiving very strong activating signal *via* a CAR or *via* potent bi- or even tri- specific antibodies and in the haplo-SCT NK infusion combination setting as well. The potential shown in especially *in vitro*, studies and the relatively high expression of classical HLA class I molecules as well as non-classical HLA-E on MM cells make it worthwhile to further explore the potential benefit of reducing inhibitory signaling *via* HLA by genetic mismatching or blocking antibodies.

## Author Contributions

All authors listed have made a substantial, direct and intellectual contribution to the work, and approved it for publication.

### Conflict of Interest Statement

The authors declare that the research was conducted in the absence of any commercial or financial relationships that could be construed as a potential conflict of interest.
